# Dietary Intake, Cost, and Affordability by Socioeconomic Group in Australia

**DOI:** 10.3390/ijerph182413315

**Published:** 2021-12-17

**Authors:** Meron Lewis, Sarah A. McNaughton, Lucie Rychetnik, Mark D. Chatfield, Amanda J. Lee

**Affiliations:** 1 School of Public Health, Faculty of Medicine, The University of Queensland, Herston 4006, Australia; m.chatfield@uq.edu.au (M.D.C.); amanda.lee@uq.edu.au (A.J.L.); 2 The Australian Prevention Partnership Centre, The Sax Institute, Glebe 2037, Australia; lucie.rychetnik@saxinstitute.org.au; 3 Institute for Physical Activity and Nutrition, School of Exercise and Nutrition Sciences, Deakin University, Geelong 3220, Australia; sarah.mcnaughton@deakin.edu.au; 4 School of Public Health, University of Sydney, Glebe 2037, Australia

**Keywords:** dietary intake, diet cost, diet affordability, low socioeconomic, Australia

## Abstract

Few Australians consume diets consistent with the Australian Dietary Guidelines. A major problem is high intake of discretionary food and drinks (those not needed for health and high in saturated fat, added sugar, salt and/or alcohol). Low socioeconomic groups (SEGs) suffer particularly poor diet-related health. Surprisingly, detailed quantitative dietary data across SEGs was lacking. Analysis of the most recent national nutrition survey data produced habitual intakes of a reference household (two adults and two children) in SEG quintiles of household income. Cost and affordability of habitual and recommended diets for the reference household were determined using methods based on the Healthy Diets Australian Standardised Affordability and Pricing protocol. Low SEGs reported significantly lower intakes of healthy food and drinks yet similarly high intakes of discretionary choices to high SEGs (435 serves/fortnight). Total habitual diets of low SEGs cost significantly less than those of high SEGs (AU$751/fortnight to AU$853/fortnight). Results confirmed low SEGs cannot afford a healthy diet. Lower intakes of healthy choices in low SEGs may help explain their higher rates of diet-related disease compared to higher SEGs. The findings can inform potential policy actions to improve affordability of healthy foods and help drive healthier diets for all Australians.

## 1. Introduction

Poor diet is a leading contributor to the burden of disease in Australia and overseas, and a major risk factor for diabetes, heart disease, and several cancers [1,2,3,4,5]. Fewer than 4% of Australians consume a healthy, equitable, and sustainable diet consistent with the Australian Dietary Guidelines (ADG) [6,7]. Further, “discretionary” food and drinks (i.e., those not needed for health and high in saturated fat, added sugar, salt, and/or alcohol) make up more than one-third of adults’ energy intake, and more than 40% of children’s energy intake [6,7].

Low socioeconomic groups (SEGs) experience higher rates of diet-related disease than the general population both globally [8,9] and in the Australian population [1,2,3]. In general, high SEGs in high-income countries are more likely to consume a healthier diet than lower SEGs [10]. This tends to hold in low- and middle-income countries (LMICs) too; however, high SEGs in urban locations in LMICs also tend to consume greater intakes of ultraprocessed foods than low SEGs [10]. Our recent systematic review of past studies of habitual dietary intake of low SEGs in Australia [11] confirmed that diet quality is usually lower in low SEGs compared to higher SEGs. However, variations in study metrics, definitions, dietary assessment methods, and granularity of data meant findings were inconsistent across studies for all reported food categories and SEG measures. Intakes of fruits and vegetables were often reported as markers of a healthy diet, but quantitative intakes of all ADG food categories by SEGs were reported rarely [11]. These data were also not readily available from the Australian Health Survey National Nutrition and Physical Activity Survey (AHS NNPAS) 2011–2013, but could be determined by detailed analysis of the individual dietary intake data [7]. The inequities of healthy eating are strongly influenced by social, economic, environmental, and commercial determinants [12]. Consideration of the relative cost of healthy and unhealthy food and drinks within the context of reported dietary intakes is particularly relevant to low SEGs [13,14]. The affordability of healthy food has been identified as a key leverage point in complex models of inequitable healthy eating, and is a product of both the cost of food and drinks and household income [15]. Affordability of healthy, equitable, and more sustainable diets is a key component of food security, which is defined as when “all people, at all times, have physical, social and economic access to sufficient, safe and nutritious food that meets their dietary needs and food preferences for an active and healthy life” [16].

The Healthy Diets Australian Standardised Affordability and Pricing (ASAP) protocol previously developed by Lee et al. compares the cost and affordability of habitual (typically unhealthy) and recommended (healthy, equitable, and more sustainable) diets for a reference household representing the general Australian population [17]. The Healthy Diets ASAP protocol includes a habitual diet pricing tool containing specific types and amounts of food and drinks based upon mean dietary intakes reported by the reference household in the AHS NNPAS [18]. The Healthy Diets ASAP protocol has been modified for specific population groups, including Aboriginal and Torres Strait Islanders [19] and low SEGs [20]. Quantitative analysis of habitual dietary intakes of different SEGs from the AHS NNPAS data could be applied to similarly modify the Healthy Diets ASAP protocol and allow calculation of the cost and affordability of habitual and recommended diets of different SEGs.

This detailed data across all ADG food categories would provide more evidence than has previously been available [11] to support targeted policies to help low-income Australians purchase and consume healthy diets and improve diet-related health. The aim of this study was to describe habitual and recommended dietary intakes, and their cost and affordability in different SEGs in Australia.

## 2. Materials and Methods

### 2.1. SEG Measure

Household income was used as the indicator of SEG as this metric reflects household resources to purchase food and is available for all subjects in the AHS NNPAS. Income quintiles were used rather than the deciles reported publicly [18], due to low sample numbers within some relevant subcategories of the AHS NNPAS (see Table 1).

### 2.2. Reference Household

The reference household in this study included two adults (female 31–50 years, male 31–50 years) and two children (boy 14–18 years, child 4–8 years). This was the same reference household described in the Healthy Diets ASAP protocol [17]. However, the included age range for children in the household was expanded from a boy 14 years and girl 8 years to a boy 14–18 years and a child 4–8 years to account for relatively low sample numbers in these subcategories of the AHS NNPAS (Table 1). 

### 2.3. Analysis of Dietary Intake Data of Different SEGs

Reported dietary intakes in the AHS NNPAS [18] were analysed by age, gender, and household income quintile to determine mean fortnightly intakes of individual reference household members. The mean reported intakes of all food and drinks for the reference household in each income quintile were then mapped to the 75 representative food and drinks of the Healthy Diets ASAP habitual diet pricing tool (Table S1) [17]. The number of serves in each food category, as defined in the ADGs [6,21,22], were then calculated. 

The ADG food categories and serve size information are included in Box 1 [6,21,22]. The energy content of the habitual diet for each SEG was analysed using the FoodWorks 9th Edition computer program [23].

Box 1 **Food and drink categories and serve size information (by weight or energy content as described in the Australian Dietary Guidelines 2013)**

- Healthy food and drink categories:
  ◦ ADG five food groups:
    ▪ Fruit (150 g/serve)
    ▪ Vegetables and legumes (75 g/serve)
    ▪ Grain (cereal) foods (mostly wholegrain) (500 kJ/serve)
    ▪ Lean meats, poultry, fish, eggs, and plant-based alternatives (550 kJ/serve)
    ▪ Milk, yoghurt, cheese, and plant-based alternatives (550 kJ/serve)
  ◦ Allowance of unsaturated oils and spreads (250 kJ/serve)
  ◦ Water (250 mL/serve)
  ◦ Artificially sweetened beverages (which are not necessary for health, but were reported within healthy food and drinks as they do not fall within the defini-tion of discretionary choices) (250 mL/serve)
- Discretionary (unhealthy) food and drink categories:
  ◦ Discretionary choices, reported in the subcategories of:
    ▪ Alcohol (600 kJ/serve)
    ▪ Takeaway foods (600 kJ/serve)
    ▪ Sugar sweetened beverages (SSBs) (600 kJ/serve)
    ▪ Discretionary choices—other (including biscuits, crisps, ice cream, con-fectionary, butter, sugar etc.) (600 kJ/serve)

### 2.4. Price Data Collection

In order to comply with the public health restrictions associated with the COVID-19 pandemic during the study period, food and drink prices were collected in June 2020 from one Statistical Area 2 (SA2) in Brisbane, Queensland, Australia (selected by convenience sampling), using the Healthy Diets ASAP web-based data collection portal [24]. 

### 2.5. Household Income Calculations for Each SEG

Fortnightly household income ranges for each SEG were calculated from available income data in the AHS NNPAS. The equivalised household income quintile ranges were adjusted for household composition, doubled to provide fortnightly income, and adjusted to account for wage increases between 2012 and 2020 [25]. 

### 2.6. Data Analysis of Cost and Affordability

Diet costs were calculated as per the methods of the Healthy Diets ASAP protocol [17], where the collected food and drink prices were applied to the 75 representative food and drinks. The mean and standard error of intake for household members in SEGs were calculated using survey weights with the Stata statistical program [26]. The mean and standard error of intakes for a household in an SEG was calculated assuming the independence of intakes of household members. Fixed-effects meta-regression models were used to perform a test for the linear trend of intakes across SEGs. *p*-values ≤ 0.05 were considered significant.

The cost of the recommended diet was calculated using the recommended diet pricing tool of the Healthy Diets ASAP protocol [17]. As healthy, equitable, and more sustainable dietary recommendations are generally similar for all Australians, the recommended diet cost was the same for each SEG.

The affordability of both habitual and recommended diets was calculated for each SEG using the calculated quintile income ranges. Diet costs were deemed unaffordable if they were 30% or more of the household disposable income [17]. If 25–29%, the household was considered to be in food stress [27].

## 3. Results

### 3.1. The Habitual Diet Reported by Different SEGs

The number of ADG food category serves in the habitual diet across SEG quintiles for a reference household (two adults, two children) per fortnight are shown in Table 2 together with the number of ADG food category serves in the recommended diet. Further details are presented in Table S2 and Figure S1.

The habitual diets provided 98–100% of the total energy intake reported by members of the household in the AHS NNPAS [18], and between 99% and 106% of the energy content of the corresponding recommended diet (Table 2). Energy contribution from discretionary food and drinks in the habitual diets was similar (*p* = 0.77) across all SEGs. The increasing energy content of habitual diets from lowest to highest SEG was due to significantly increasing contributions from healthy food and drinks (*p* = 0.001).

Likewise, the total reported number of serves of healthy food and drinks increased significantly (*p* < 0.001) from SEG quintile 1 (lowest income) to SEG quintile 5 (highest income), whereas the high total number of serves of discretionary food and drinks was similar across all SEGs (*p* = 0.71).

For all SEGs, the number of serves of each of the ADG five food groups in the habitual diet were much less than the recommended number of serves (Table 2). The reported number of serves increased significantly from SEG quintile 1 (lowest income) to SEG quintile 5 (highest income) for: fruit (*p* = 0.048); grain (cereal) foods, mostly wholegrain (*p* = 0.023); and lean meats, poultry, fish, eggs, and plant-based alternatives (*p* = 0.002). The number of serves for vegetables and legumes tended to increase from SEG quintile 1 to SEG quintile 5, although the increase was not significant (*p* = 0.085). The reported number of serves of artificially sweetened beverages increased significantly (*p* = 0.002) from SEG quintile 1 (lowest income) to SEG quintile 5 (highest income). Conversely, the reported number of serves for unsaturated oils and spreads tended to decrease (*p* = 0.078) from SEG quintile 1 (lowest income) to SEG quintile 5 (highest income). 

For the components of the discretionary items, the reported number of serves for alcoholic drinks increased significantly (*p* = 0.002) from SEG quintile 1 (lowest income) to SEG quintile 5 (highest income). The reported number of serves of SSBs and, to a lesser extent takeaway foods, tended to decrease from SEG quintile 1 (lowest income) to SEG quintile 5 (highest income), although these trends was not significant (*p* = 0.34, 0.17, respectively) (Table 2). Similarly, there was a slight but non-significant (*p* = 0.12) trend for the reported number of serves of discretionary choices—other to increase from SEG quintile 1 to SEG quintile 5. 

### 3.2. Cost of Habitual Diets in Different SEGs and the Recommended Diet 

**Figure 1:**
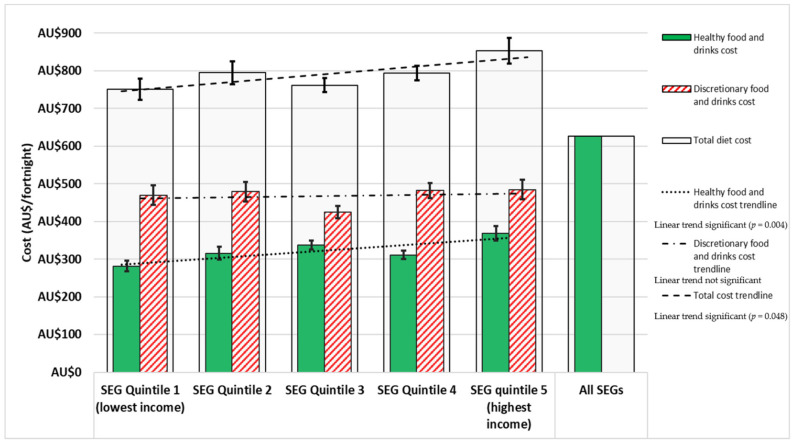
Cost of the habitual diet per fortnight for different SEGs, and cost of the recommended diet per fortnight, for a reference household (two adults, two children), including total diet cost, healthy and discretionary food and drink component costs. Error bars denote standard errors.

**Figure 2:**
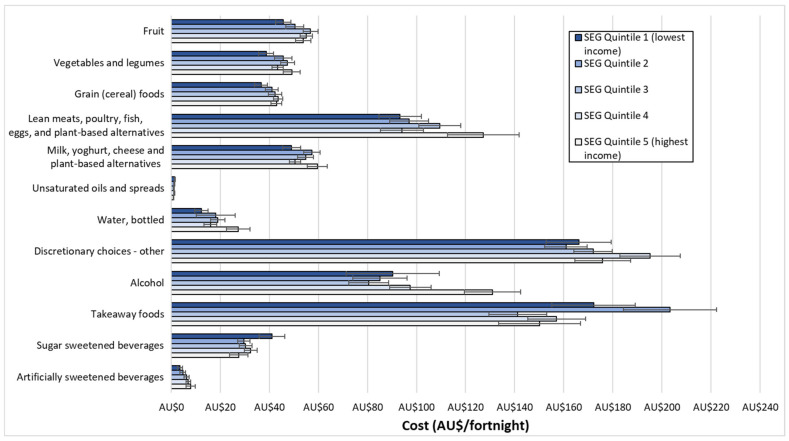
Habitual diet food category costs for each SEG for a reference household (two adults, two children). Error bars denote standard errors.

Figure 1 shows total cost of the habitual diet for the reference household per fortnight for each SEG, and the cost of the recommended diet for the reference household per fortnight. Additionally shown are the cost components for healthy and discretionary food and drinks within each diet. Figure 2 shows the habitual diet food category costs for each SEG. Detailed costs of habitual diets of different SEGs and the recommended diet, by food categories, are presented in Table S3. 

As could be expected, costs followed the patterns of dietary intake, providing relevant insights into dietary impacts across SEGs. For all SEGs, the cost of the habitual diet was greater than the cost of the recommended diet by 17% to 27% (AU$124 to AU$227 per fortnight) (Figure 1). The cost of the habitual diet for the highest SEG was significantly more expensive (AU$103 per fortnight) compared to the lowest SEG (*p* = 0.021). The total cost of the habitual diet increased from lowest to highest SEG (*p* = 0.048).

### 3.3. Cost of Discretionary Choices in the Habitual Diet 

In all SEGs, the reference household spent the majority of their food budget on discretionary items: varying between 56% and 63% of the total habitual diet costs (Figure 1). There was no significant linear trend (*p* = 0.56) in the overall cost of discretionary food and drinks in the habitual diet of different SEGs. However, the cost of alcohol increased significantly from low to high SEG (*p* = 0.004), and the cost of SSBs tended (*p* = 0.34) to decrease from low to high SEG (Figure 2).

### 3.4. Cost of Healthy Choices in the Habitual Diet

The reference household in different SEGs spent between 37% and 44% of the total food budget on healthy food and drinks (Figure 1). There was a significant increase (*p* = 0.004) in the cost of healthy food and drinks combined in the habitual diet from low to high SEG, and for the subgroups of fruit (*p* = 0.033), bottled water (*p* = 0.032), and artificially sweetened beverages (*p* = 0.002) (Figure 2). 

### 3.5. Affordability of Habitual and Recommended Diets

The range of fortnightly gross household income of the representative household and the proportion of household income required to be spent on habitual and recommended diets in each SEG quintile are shown in Table 3. Both diets are unaffordable for households in the lowest SEG. For households in SEG quintile 2, the cost of the habitual diet is either unaffordable or causes ‘food stress’, and the cost of the recommended diet causes ‘food stress’. For households in SEG quintiles 3, 4, and 5, both diets were affordable and did not cause ‘food stress’.

## 4. Discussion

### 4.1. Summary

Low SEG households reported habitual diets that included significantly lower amounts of healthy, yet similar amounts of discretionary, food and drinks than higher SEG households. When the diets were costed, the habitual diets of low SEGs were of significantly lower cost than high SEGs, due to a trend of increasing cost of healthy food and drinks from the lowest to highest SEG quintile for the reference household but similar costs of discretionary food and drinks across quintiles. Analysis of more granular food category intakes and costs showed additional differences between SEGs. Recommended diets were unaffordable for the lowest SEG, and stressful to afford for the second lowest SEG.

### 4.2. Diet

Analysis of reported food and drinks in the AHS NNPAS of a reference household revealed that intake of most healthy food and drinks increased from lowest to highest SEGs, while intake of discretionary food and drinks was similar across all SEGs. However, within the discretionary choices subcategories, intake of SSBs tended to decrease from lower to higher SEGs, and intake of alcohol increased from lower to higher SEGs. The energy content of each habitual diet was within 2% of the energy content of corresponding reported dietary intakes of the different SEG quintiles in the AHS NNPAS, supporting face validity of the constructed habitual diets [18]. 

Food choice is influenced by many complex social barriers, which affect access to resources [8,11,28]. The different intakes of healthy food and drinks in different SEGs may relate to different food preparation time and resources, differing perceptions that healthy foods are too expensive, and higher promotion of unhealthy foods in the food environment in low SEG areas [12,29,30,31]. 

The intake of artificially sweetened beverages increased from low SEGs to higher SEGs, while, although not significant, intake of SSBs decreased from low SEGs to higher SEGs. These trends provide insights that may be useful to understand the impact of an SSB tax and provide context for its application. For example, it has been postulated that a “SSB tax”, although potentially regressive (i.e., having greater impact on low SEGs), could provide greater health benefits for low SEGs than the rest of the population [32]. 

Alcohol intake has rarely been included in previous analyses of dietary intake in Australia, including in studies of reported dietary intakes of different SEGs [11]. The increased alcohol intake from lower to higher SEGs seen in this study is consistent with previous findings of reported intakes in studies of alcohol consumption specifically [33,34]. The reference household did not include a representative from the 18–29 years age group who consume the highest quantities of alcohol in Australia [33,34]. Results show alcohol contributes 3–5% of energy intake and costs 12–15% of the food budget for the reference household in different SEGs, thus impacting both health and food affordability. These findings confirm that alcohol intake should be considered in nutrition policies [35], including those supporting equity. 

The habitual diets are based upon reported intakes in the most recent national food and nutrition survey, the AHS NNPAS of 2011–2013, and it is possible that dietary intake patterns have altered over the last 10 years. Limited changes were noted between the 1995 National Nutrition Survey and the AHS NNPAS 2011–2013 [36]; however, changes may have occurred subsequently. This is particularly the case as changes in the food environment since 2013, such as the rise in online food delivery options [37,38] and the impact of the COVID-19 pandemic [39,40,41], may have influenced dietary intakes. 

### 4.3. Cost

Habitual diets were found to be more expensive than recommended diets, consistent with other studies applying the original Healthy Diets ASAP protocol [19,42,43]. This is partially due to the exemption of basic healthy foods, but not discretionary choices, from the 10% Goods and Services Tax (GST) in Australia, so that a higher amount of GST is payable on habitual diets compared to recommended diets [44,45]. The effect of the introduction of the GST in 2000 on national dietary intakes has not been reported, due to infrequent Australian dietary surveys [46]. Increasing habitual diet cost across SEG quintiles is also consistent with the results of the ABS Household Expenditure Survey, which found low-income households spent less on food than higher-income households [47].

More granular analysis of the costs of habitual diets showed that the cost of healthy food and drinks, rather than discretionary food and drinks, is the main factor contributing to dietary cost differences across SEG quintiles. There was a significant increase in the cost of all healthy food and drink categories from the lowest to the highest SEG. The overall cost of discretionary food and drinks was similar across different SEGs, although the costs of SSBs tended to decrease, and costs of alcohol increased, from lowest to highest SEGs. 

The indicated trend, although not significant, of decreasing cost of takeaway foods from lower to higher SEGs contrasts with household expenditure surveys showing low SEGs spend less on ‘meals out and fast foods’ than higher SEGs [47]. However, household expenditure surveys solely reflect the purchase amount, rather than the type, quantity, and nutritional quality of food being purchased. The results of the current study, based on reported dietary intakes, tend to support previous studies that suggest that when low SEGs consume food prepared outside the home, they tend to purchase ‘fast food’ rather than healthier meals, such as that available in restaurants [48,49].

### 4.4. Affordability

An affordable diet has been defined generally as one where food and drink purchases require 30% or less of the household’s disposable income [17]. Our results show that recommended diets were not affordable for the lowest SEG, and their cost caused ‘food stress’ for SEG quintile 2 households. In addition, there may be circumstances where other expenses, such as housing (mortgage and rental), transport, and education costs, are so high that less than 30% of income is available for food purchasing [50,51,52]. These economic pressures can be heavy, for example, in 2017, 35% of low-income families with children spent more than 30% of their household income on housing [53]. Additionally, the most recent household expenditure survey in 2015 found 15% of all households, and 32% of low-income households, were in ‘financial stress’ [47]. In such circumstances, the household food budget, being more flexible than fixed expenses, such as rent and utility bills, may be reduced to compensate [54]. Therefore, the findings of this study may underestimate the financial challenges faced by low-income households when purchasing food.

### 4.5. Strengths and Limitations

The use of the standardized Healthy Diets ASAP protocol as a framework for analysis of habitual dietary intakes of different SEG households provides an example of granular, whole of diet analysis to produce policy-relevant data. There is scope for similar methods to be developed in other countries, which would allow inter-country comparison and the potential for cross-seeding of policy action [13].

Household income was selected as the measure of SEG as it reflected current household resources for food purchases, even if a recent lifestyle change had occurred, such as job loss/gain or family separation. Other SEG measures used in dietary intake studies in Australia included less changeable measures of education, occupation, disadvantage level of the residential area, and/or combinations thereof, although household income has been most commonly used [11]. Some previous studies found differences in SEG gradients of dietary intake using different measures of SEG [55,56,57]; however, education level and occupation are not available in the AHS NNPAS for all reference household members [58].

Assessment of dietary intake by 24-hour recall (as used in the AHS NNPAS) is known to be biased by social desirability, particularly in women, overweight/obese persons, and low SEGs [58,59]. The ABS has estimated that average energy intakes reported in the AHS NNPAS may be understated by 17% in males and 21% in females [58]. Further, social desirability bias tends to inflate healthy food intakes and under-report less healthy intakes [59]. No adjustments were made to the dietary intake analysis to account for such likely misreporting. This may have resulted in overestimation of the intake and costs of healthy foods, and underestimation of the intake and costs of discretionary foods, particularly in the lower SEGs. 

There are some inherent limitations of determining diet cost using the Healthy Diets ASAP protocol, which are applicable to this study and have been detailed elsewhere [17]. These include the assumptions that food is equitably shared among all household members, that there is minimal food wastage, and that home production of food is minimal. A potential further limitation relates to the price data, which was collected from one SA2 area. This may limit the generalisability of the results. However, no significant difference in diet cost across areas of varying socioeconomic status (within major cities) was found in a recent application of the Healthy Diets ASAP protocol [28].

### 4.6. Recommendations

The analysis has shown differences in reported dietary intakes across SEGs at a granular, ADG food category level that have not been quantified previously. These data confirm the need for an equity lens in nutrition policy and practice, including the development of national policy tools, such as food-based dietary guidelines. 

The results of the study also suggest that measures other than fiscal and economic policies should be considered to incentivise the purchase of healthy food and drinks. The placement, and price promotion, of healthy food and drinks together with restrictions on the availability, placement, and promotion of unhealthy food and drinks may encourage the purchase of the former in place of the latter [60].

The exemption of GST on basic healthy foods in Australia helps keep the cost of recommended diets lower, but results suggest this does not go far enough; increasing the GST rate on unhealthy food and drinks would further increase the cost differential between habitual and recommended diets. Modelling has shown that an increase of the GST rate on unhealthy foods to 20% would increase the relative affordability of recommended diets to habitual diets by another 9% and help drive healthier, more equitable, and sustainable diets [61].

One clear option to improve diet affordability is to increase household income. This has been exemplified by a recent natural experiment. During the COVID-19 pandemic, the Australian Government implemented economic stimulus measures to combat the sudden increase in un- and under-employment. This resulted in increased income for many low-income and welfare-dependent households and improved the affordability of a healthy diet [62]. Whilst these economic measures were only of short duration, this real-life example suggests the potential health impact of increasing longer-term income support for low SEGs. A survey following the implementation of the economic stimulus measures found 83% of welfare-dependent people reported eating more regularly, and healthier, than before the pandemic [63]. Implementation of these policy actions would increase economic access to healthy, equitable, and more sustainable diets and therefore help improve food security and diet-related health in low SEGs in Australia [64,65].

## 5. Conclusions

For the first time, this study describes habitual dietary intakes, costs, and affordability by SEG in Australia. It also provides granular, quantitative data across all ADG food categories, which have been absent from the literature [11]. The intake of discretionary food and drinks was similar across SEGs, while the intake of healthy food and drinks decreased from high to low SEGs. Insights into varying intakes within subcategories of discretionary food and drinks provides further data to inform policies specific to low SEGs. Results confirm that affording a healthy diet is problematic for low SEGs. This highlights the need to keep the price of healthy food as low as possible, both in absolute terms and relative to the price of unhealthy food. It also highlights the need to ensure low SEG households have sufficient income so that healthy and sustainable diets are affordable for all Australians. This study provides valuable evidence to support the development of targeted policies to help low-income households purchase and consume healthy diets, and improve diet-related health.

## Figures and Tables

**Table 1:** Participant numbers in subcategories in the ABS NNPAS.

	All Income Levels	SEG Quintile 1 (Lowest Income)	SEG Quintile 2	SEG Quintile 3	SEG Quintile 4	SEG Quintile 5 (Highest Income)
Male 31–50, (*n*)	1669	151	208	329	417	464
Female 31–50, (*n*)	1896	258	293	355	429	425
Boy 14, (*n*)	72	10	14	13	17	8
Boy 14–18, (*n*)	403	49	67	69	91	54
Girl 8, (*n*)	67	9	14	19	12	6
Child 4–8, (*n*)	789	120	156	180	155	112

**Table 2:** Energy (MJ/day) and ADG food intake (serves per fortnight) of habitual diets across SEGs, and recommended diet, for a reference household (two adult, two children) per fortnight.

			Habitual Diet						Recommended Diet
			SEG Quintile 1 (Lowest Income)	SEG Quintile 2	SEG Quintile 3	SEG Quintile 4	SEG Quintile 5 (Highest Income)	*p*-Value of Linear Trend	
All Healthy food and drinks, Energy (MJ/day), Mean ± standard error			13.9 ± 0.5	15.3 ± 0.5	15.6 ± 0.5	15.2 ± 0.4	16.3 ± 0.4	0.001 **	33.0
All Discretionary (unhealthy) food and drinks, Energy (MJ/day), Mean ± standard error			18.6 ± 0.8	18.9 ± 0.8	17.9 ± 0.6	19.2 ± 0.6	18.6 ± 0.9	0.77	0
Total Diet, Energy (MJ/day), Mean ± standard error			32.5 ± 0.9	34.2 ± 0.9	33.5 ± 0.7	34.4 ± 0.6	34.9 ± 1.0	0.085	33.0
All Healthy food and drinks, Serves/fortnight mean ± standard error			496 ± 14	551 ± 18	567 ± 13	549 ± 12	599 ± 17	<0.001 **	1215
	ADG Five food groups	Fruit (150 g/serve)	64 ± 4	68 ± 4	77 ± 3	74 ± 4	74 ± 5	0.048 **	109
		Vegetables and Legumes (75 g/serve)	95 ± 6	115 ± 11	111 ± 6	104 ± 6	118 ± 8	0.085	302
		Grain (cereal) foods, mostly wholegrain (500 kJ/serve)	145 ± 7	150 ± 7	164 ± 8	164 ± 7	163 ± 9	0.023 **	329
		Lean Meats, Poultry, Fish, Eggs, and Plant-based alternatives (550 kJ/serve)	66 ± 5	80 ± 5	81 ± 4	78 ± 4	96 ± 6	0.002 **	169
		Milk, Yoghurt, Cheese and Plant-based Alternatives (550 kJ/serve)	83 ± 5	91 ± 5	86 ± 4	81 ± 4	89 ± 6	0.76	158
	Unsaturated Oils and Spreads Allowance (250 kJ/serve)		24 ± 4	20 ± 2	16 ± 2	19 ± 3	15 ± 2	0.078	127
	Bottled Water (250 mL/serve)		14 ± 3	21 ± 9	22 ± 3	18 ± 3	31 ± 6	0.032 **	21
	Artificially sweetened beverages (250 mL/serve)		6 ± 2	8 ± 2	10 ± 2	11 ± 2	13 ± 3	0.002 **	0
All Discretionary (unhealthy) food and drinks Serves/fortnight, mean ± standard error			435 ± 20	441 ± 17	417 ± 14	449 ± 14	434 ± 20	0.71	0
	Discretionary Choices (600 kJ/serve)	Alcoholic Drinks	25 ± 5	23 ± 3	23 ± 2	27 ± 2	37 ± 3	0.002 **	0
		Takeaway foods	119 ± 12	131 ± 12	98 ± 8	106 ± 8	106 ± 12	0.17	0
		Sugar-sweetened beverages	46 ± 6	33 ± 3	34 ± 3	36 ± 3	31 ± 4	0.34	0
		Discretionary Choices—other	245 ± 14	254 ± 12	264 ± 11	279 ± 10	260 ± 15	0.12	0

** *p* ≤ 0.05.

**Table 3:** Gross household income, habitual and recommended diet cost, and affordability per fortnight in each SEG for a reference household.

SEG Quintile	Household Income per Fortnight (AU$)	Habitual Diet		Recommended Diet	
		Cost Per Fortnight (AU$)	Affordability (% of Household Income)	Cost Per Fortnight (AU$)	Affordability (% of Household Income)
1 (lowest income)	<$2129	$751	>35%	$627	>29%
2	$2129–$3413	$795	23–35%	$627	18–29%
3	$3413–$5125	$761	15–23%	$627	12–18%
4	$5125–$7688	$793	10–15%	$627	8–12%
5 (highest income)	>$7688	$853	<10%	$627	<8%

## Data Availability

The data presented in this study are available in this article and its Supplementary Materials.

## Supplementary Materials

The following are available online at https://www.mdpi.com/article/10.3390/ijerph182413315/s1, Table S1: Concordance between Australian Health Survey National Nutrition and Physical Activity Survey food and drinks and Healthy Diets ASAP food and drinks, Table S2: Composition of the habitual diet of each SEG, and the recommended diet, for the reference household (two adults, two children), Table S3: Habitual diet costs of each SEG and recommended diet costs, for the representative household (two adults, two children), Figure S1: Habitual diet serves per fortnight (as per Australian Dietary Guidelines) for each SEG for a reference household (two adults, two children).

## References

[1] Australian Institute of Health and Welfare (2016). Australia’s Health 2016-Health of Population Groups: Health across Socioeconomic Groups. https://www.aihw.gov.au/reports/australias-health/australias-health-2016/contents/chapter-5-health-of-population-groups.

[2] Stringhini S., Carmeli C., Jokela M., Avendaño M., Muennig P., Guida F., Ricceri F., d’Errico A., Barros H., Bochud M. (2017). Socioeconomic status and the 25 × 25 risk factors as determinants of premature mortality: A multicohort study and meta-analysis of 1.7 million men and women. Lancet.

[3] Harris B., Fetherston H., Calder R. (2017). Australia’s Health Tracker by Socio-Economic Status 2017.

[4] Murray C.J.L., Aravkin A.Y., Zheng P., Abbafati C., Abbas K.M., Abbasi-Kangevari M., Abd-Allah F., Abdelalim A., Abdollahi M., Abdollahpour I. (2020). Global burden of 87 risk factors in 204 countries and territories, 1990–2019: A systematic analysis for the Global Burden of Disease Study 2019. Lancet.

[5] Australian Institute of Health and Welfare (2018). Australian Burden of Disease Study 2018-Key Findings. https://www.aihw.gov.au/reports/burden-of-disease/burden-of-disease-study-2018-key-findings/contents/about.

[6] National Health and Medical Research Council (2013). Australian Dietary Guidelines-Providing the Scientific Evidence for Healthier Australian Diets.

[7] Australian Bureau of Statistics (2014). 4364.0.55.007-Australian Health Survey: Nutrition First Results-Foods and Nutrients, 2011–2012. http://www.abs.gov.au/AUSSTATS/abs@.nsf/DetailsPage/4364.0.55.0072011-12?OpenDocument.

[8] Marmot M., Allen J., Bell R., Bloomer E., Goldblatt P. (2012). WHO European review of social determinants of health and the health divide. Lancet.

[9] Sawyer A.D.M., van Lenthe F., Kamphuis C.B.M., Terragni L., Roos G., Poelman M.P., Nicolaou M., Waterlander W., Djojosoeparto S.K., Scheidmeir M. (2021). Dynamics of the complex food environment underlying dietary intake in low-income groups: A systems map of associations extracted from a systematic umbrella literature review. Int. J. Behav. Nutr. Phys. Act..

[10] Mayén A.L., Marques-Vidal P., Paccaud F., Bovet P., Stringhini S. (2014). Socioeconomic determinants of dietary patterns in low- and middle-income countries: A systematic review. Am. J. Clin. Nutr..

[11] Lewis M., Lee A.J. (2021). Dietary inequity? A systematic scoping review of dietary intake in low socio-economic groups compared with high socio-economic groups in Australia. Public Health Nutr..

[12] Zorbas C., Palermo C., Chung A., Iguacel I., Peeters A., Bennett R., Backholer K. (2018). Factors perceived to influence healthy eating: A systematic review and meta-ethnographic synthesis of the literature. Nutr. Rev..

[13] Lee A., Mhurchu C.N., Sacks G., Swinburn B., Snowdon W., Vandevijvere S., Hawkes C., L’Abbe M., Rayner M., Sanders D. (2013). Monitoring the price and affordability of foods and diets globally. Obes. Rev..

[14] Monsivais P., Aggarwal A., Drewnowski A. (2012). Are socio-economic disparities in diet quality explained by diet cost?. J. Epidemiol. Community Health.

[15] Friel S., Pescud M., Malbon E., Lee A., Carter R., Greenfield J., Cobcroft M., Potter J., Rychetnik L., Meertens B. (2017). Using systems science to understand the determinants of inequities in healthy eating. PLoS ONE.

[16] FAO (2002). The State of Food Insecurity in the World 2001.

[17] Lee A.J., Kane S., Lewis M., Good E., Pollard C.M., Landrigan T.J., Dick M. (2018). Healthy diets ASAP-Australian Standardised Affordability and Pricing methods protocol. Nutr. J..

[18] Australian Bureau of Statistics (2013). 4324.0.55.002 Microdata: Australian Health Survey: Nutrition and Physical Activity, 2011–2012. http://www.abs.gov.au/ausstats/abs@.nsf/PrimaryMainFeatures/4324.0.55.002?OpenDocument.

[19] Lee A., Lewis M. (2018). Testing the price of healthy and current diets in remote Aboriginal communities to improve food security: Development of the Aboriginal and Torres Strait Islander Healthy Diets ASAP (Australian Standardised Affordability and Pricing) methods. Int. J. Environ. Res. Public Health.

[20] Lewis M., McNaughton S.A., Rychetnik L., Lee A.J. (2021). Cost and Affordability of Healthy, Equitable and Sustainable Diets in Low Socioeconomic Groups in Australia. Nutrients.

[21] National Health and Medical Research Council (2013). Eat for Health Educator Guide.

[22] National Health and Medical Research Council (2011). A Modelling System to Inform the Revision of the Australian Guide to Healthy Eating.

[23] Xyris Software (Australia) Pty Ltd (2019). FoodWorks Professional 9th Edition.

[24] Lee A.J., Lewis M., Goodwin S. (2020). Healthy Diets ASAP Portal. https://healthydiets.azurewebsites.net/.

[25] Australian Bureau of Statistics (2020). 6345.0-Wage Price Index, Australia. https://www.abs.gov.au/statistics/economy/price-indexes-and-inflation/wage-price-index-australia/latest-release.

[26] StataCorp (2019). Stata 16.0.

[27] Landrigan T.J., Kerr D.A., Dhaliwal S.S., Pollard C.M. (2018). Protocol for the development of a food stress index to identify households most at risk of food insecurity in Western Australia. Int. J. Env. Res. Public Health.

[28] Lee A., Patay D., Herron L.-M., Parnell Harrison E., Lewis M. (2021). Affordability of current, and healthy, more equitable, sustainable diets by area of socioeconomic disadvantage and remoteness in Queensland: Insights into food choice. Int. J. Equity Health.

[29] Ball K., Crawford D., Mishra G. (2007). Socio-economic inequalities in women’s fruit and vegetable intakes: A multilevel study of individual, social and environmental mediators. Public Health Nutr..

[30] Burns C. (2008). The vulnerable and the disadvantaged. Aust. Econ. Rev..

[31] Thornton L.E., Lamb K.E., Ball K. (2016). Fast food restaurant locations according to socioeconomic disadvantage, urban–regional locality, and schools within Victoria, Australia. SSM-Popul. Health.

[32] Backholer K., Sarink D., Beauchamp A., Keating C., Loh V., Ball K., Martin J., Peeters A. (2016). The impact of a tax on sugar-sweetened beverages according to socio-economic position: A systematic review of the evidence. Public Health Nutr..

[33] Roche A., Kostadinov V., Fischer J., Nicholas R., Victorian Health Promotion Foundation (2015). Evidence Review: The Social Determinants of Inequities in Alcohol Consumption and Alcohol-Related Health Outcomes.

[34] Commonwealth of Australia, National Health and Medical Research Council (2020). Australian Guidelines to Reduce Health Risks from Drinking Alcohol.

[35] National Heart Foundation of Australia (2021). Position Statement: Alcohol and Heart Health.

[36] Olstad D.L., Leech R.M., Livingstone K.M., Ball K., Thomas B., Potter J., Cleanthous X., Reynolds R., McNaughton S.A. (2018). Are dietary inequalities among Australian adults changing?. a nationally representative analysis of dietary change according to socioeconomic position between 1995 and 2011–13. Int. J. Behav. Nutr. Phys. Act..

[37] Dana L.M., Hart E., McAleese A., Bastable A., Pettigrew S. (2021). Factors associated with ordering food via online meal ordering services. Public Health Nutr..

[38] Roy Morgan Research (2021). Meal delivery services Uber Eats, Menulog, Deliveroo and DoorDash Experienced Rapid Growth during 2020-a Year of Lockdowns & Work from Home. http://www.roymorgan.com/findings/8713-food-delivery-services-may-2021-202105280627.

[39] Bakaloudi D.R., Jeyakumar D.T., Jayawardena R., Chourdakis M. (2021). The impact of COVID-19 lockdown on snacking habits, fast-food and alcohol consumption: A systematic review of the evidence. Clin. Nutr..

[40] Theobald C., White A. (2021). British Nutrition Foundation Healthy Eating Week 2020-insights into the effect of COVID-19 on eating and activity habits of adults and children in the UK. Nutr. Bull..

[41] Koltai J., Toffolutti V., McKee M., Stuckler D. (2021). Prevalence and changes in food-related hardships by socioeconomic and demographic groups during the COVID-19 pandemic in the UK: A longitudinal panel study. Lancet Reg. Health Eur..

[42] Lee A.J., Kane S., Herron L.-M., Matsuyama M., Lewis M. (2020). A tale of two cities: The cost, price-differential and affordability of current and healthy diets in Sydney and Canberra, Australia. Int. J. Behav. Nutr. Phys. Act..

[43] Love P., Whelan J., Bell C., Grainger F., Russell C., Lewis M., Lee A. (2018). Healthy diets in rural Victoria—Cheaper than unhealthy alternatives, yet unaffordable. Int. J. Environ. Res. Public Health.

[44] Australian Taxation Office (2020). GST-Free Sales. https://www.ato.gov.au/business/gst/when-to-charge-gst-(and-when-not-to)/gst-free-sales/.

[45] Landrigan T.J., Kerr D.A., Dhaliwal S.S., Savage V., Pollard C.M. (2017). Removing the Australian tax exemption on healthy food adds food stress to families vulnerable to poor nutrition. Aust. N. Z. J. Public Health.

[46] Seal J. (2004). Monitoring the price and availability of healthy food—time for a national approach?. Nutr. Diet..

[47] Australian Bureau of Statistics (2017). 6530.0-Household Expenditure Survey, Australia: Summary of Results, 2015–2016. https://www.abs.gov.au/statistics/economy/finance/household-expenditure-survey-australia-summary-results/latest-release.

[48] Thornton L.E., Bentley R.J., Kavanagh A.M. (2011). Individual and area-level socioeconomic associations with fast food purchasing. J. Epidemiol. Community Health.

[49] Miura K., Giskes K., Turrell G. (2011). Socio-economic differences in takeaway food consumption among adults. Public Health Nutr..

[50] Victorian Health Promotion Foundation (2016). Too Little and too Much: Exploring the Paradox of Food Insecurity and Obesity in Disadvantaged Populations: Research Highlights.

[51] Zorbas C., Browne J., Chung A., Peeters A., Booth S., Pollard C., Allender S., Hawkes C., Isaacs A., Backholer K. Lifting the Silence on Lived Experiences with Food and Low Incomes during COVID-19. Proceedings of the ANZOS Annual Scientific Meeting 2021.

[52] Temple J.B., Booth S., Pollard C.M. (2019). Social assistance payments and food insecurity in Australia: Evidence from the Household Expenditure Survey. Int. J. Env. Res. Public Health.

[53] Australian Bureau of Statistics (2019). Housing Occupancy and Costs, 2017–2018. https://www.abs.gov.au/statistics/people/housing/housing-occupancy-and-costs/latest-release#housing-affordability.

[54] Booth S., Smith A. (2001). Food security and poverty in Australia—challenges for dietitians. Aust. J. Nutr. Diet..

[55] Livingstone K.M., Olstad D.L., Leech R.M., Ball K., Meertens B., Potter J., Cleanthous X., Reynolds R., McNaughton S.A. (2017). Socioeconomic inequities in diet quality and nutrient intakes among Australian adults: Findings from a nationally representative cross-sectional study. Nutrients.

[56] Turrell G., Hewitt B., Patterson C., Oldenburg B. (2003). Measuring socio-economic position in dietary research: Is choice of socio-economic indicator important?. Public Health Nutr..

[57] Zarnowiecki D., Ball K., Parletta N., Dollman J. (2014). Describing socioeconomic gradients in children’s diets-does the socioeconomic indicator used matter?. Int. J. Behav. Nutr. Phys. Act..

[58] Australian Bureau of Statistics (2014). 4364.0.55.001-Australian Health Survey: Users’ Guide, 2011–2013. https://www.abs.gov.au/ausstats/abs@.nsf/Lookup/5209F2553DE3B084CA257BBB0014D160?opendocument.

[59] Giskes K., Turrell G., Patterson C., Newman B. (2002). Socio-economic differences in fruit and vegetable consumption among Australian adolescents and adults. Public Health Nutr..

[60] Sacks G., Schultz S., Grigsby-Duffy L., Robinson E., Orellana L., Marshall J., Cameron A.J. (2020). Inside Our Supermarkets: Assessment of the Healthiness of Australian Supermarkets.

[61] Lee A.J., Kane S., Ramsey R., Good E., Dick M. (2016). Testing the price and affordability of healthy and current (unhealthy) diets and the potential impacts of policy change in Australia. BMC Public Health.

[62] Lewis M., Lee A.J. (2020). Affording health during the COVID-19 pandemic and associated economic downturn. Aust. N. Z. J. Public Health.

[63] Australian Council of Social Services (2020). Survey of 955 People Receiving the New Rate of Jobseeker and Other Allowances.

[64] Bowden M., Child Family Community Australia (2020). Understanding Food Insecurity in Australia. Information Exchange.

[65] Butcher L.M., Ryan M.M., O’Sullivan T.A., Lo J., Devine A. (2018). What drives food insecurity in Western Australia? How the perceptions of people at risk differ to those of stakeholders. Nutrients.

