# Supplementary material for: Dietary Intake, Cost, and Affordability by Socioeconomic Group in Australia
Source: Int J Environ Res Public Health. 2021 Dec 17;18(24):13315. doi: 10.3390/ijerph182413315 (PMC8703846; doi:10.3390/ijerph182413315)

Figure S1: Habitual diet serves per fortnight (as per Australian Dietary Guidelines) for each SEG for a reference household (two adults, two children). Errors bars denote standard errors reflecting variation of dietary intakes.

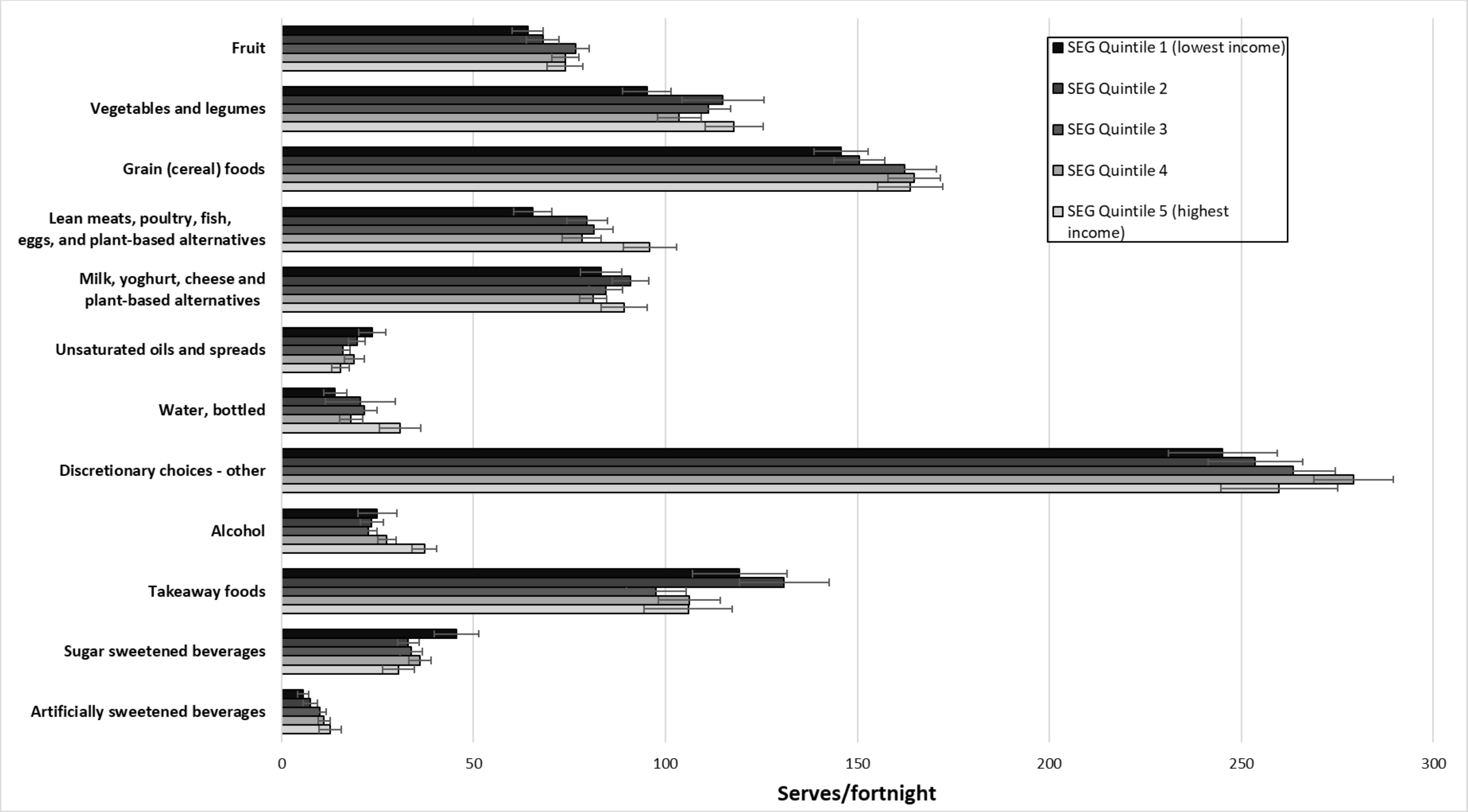

Supplement: Supplementary file 1 [file ijerph-18-13315-s001.zip › Additional Figure 1.pdf]
