# Supplementary material for: Dietary Intake, Cost, and Affordability by Socioeconomic Group in Australia
Source: Int J Environ Res Public Health. 2021 Dec 17;18(24):13315. doi: 10.3390/ijerph182413315 (PMC8703846; doi:10.3390/ijerph182413315)
Supplement: Supplementary file 1 [file ijerph-18-13315-s001.zip › Additional table 1.pdf]

Table S1: Concordance between ABS NNPAS food codes and food and drink items of the Healthy Diets ASAP habitual diet pricing tool

Food Group	Food and drink items of the Low SEG Healthy Diets ASAP habitual diet pricing tool	AHS NNPAS Code	Item	Proportion of weight included (100% if blank)
**Water**	Water, bottled	11702	Purchased packaged water including mineral water	
		11703	Purchased packaged water, fortified	
**Fruit**	Apples	16101	Apples	
		16102	Apples, commercially sterile	
		16103	Pears	
		16104	Pears, commercially sterile	
		16105	Other pome fruit	
	Bananas	16501	Bananas	
	Oranges	16201	Berry fruit	
		16202	Berry fruit, commercially sterile	
		16301	Oranges	
		16302	Lemons and limes	
		16303	Other citrus fruit	
		16304	Citrus fruit, commercially sterile	
	Fruit salad, canned in juice	16401	Peaches and nectarines	
		16402	Peaches and nectarines, commercially sterile	
		16403	Other stone fruit	
		16404	Other stone fruit, commercially sterile	
		16502	Pineapples	
		16503	Other tropical and subtropical fruit, edible peel	
		16504	Other tropical and subtropical fruit, inedible peel	
		16505	Tropical and subtropical fruit, commercially sterile	
		16601	Other fruit	
		16701	Mixtures of two or more groups of fruit	
		16702	Mixtures of two or more groups of fruit, commercially sterile	
		16801	Dried vine fruit	
		16802	Other dried fruit including mixed dried fruit	
		16804	Preserved fruit	
		16901	Mixed dishes where fruit is the major component	
		32301	Infant fruit and fruit-based desserts	
**Vegetables & Legumes**	Potato, loose	24101	Potatoes	
		24103	Potato mixed dishes	
	Sweetcorn, canned	24704	Sweetcorn	
	Broccoli, loose	24201	Cabbage and similar brassica vegetables	
		24202	Broccoli, broccolini and cauliflower	
		24402	Stalk vegetables	
	White cabbage, loose	24401	Leaf vegetables	
		24403	Herbs, fresh	
		24404	Seaweeds	
	Iceberg lettuce, whole	24904	Salads, vegetable based	
		24905	Salads, vegetable based, added meat, fish or eggs	50%
	Carrot, loose	24301	Carrots	
		24302	Other root vegetables	
	Pumpkin	24701	Pumpkin	
		24702	Squash and zucchini	
		24703	Mushrooms	
	Four bean mix, canned	25101	Mature legumes and pulses	
		25102	Mature legumes and pulses, commercially sterile	
	Diced tomatoes, canned	24602	Tomato products	
		18708	Pork dishes with gravy, sauce or vegetables	15%
		18709	Pork dishes, added pasta, noodles or rice	15%
		18901	Poultry dishes, with gravy, sauce or vegetables	15%
		18902	Poultry dishes, with gravy, sauce or vegetables, added pasta, noodles or rice	15%
		18801	Sausage dishes with gravy, sauce or vegetables	15%
		18802	Sausage dishes with gravy, sauce or vegetables, added pasta, noodles or rice	15%
	Onion, loose	24802	Onion, leek and garlic	
	Tomatoes, loose	24601	Tomato	
	Frozen mixed vegetables	24705	Other fruiting vegetables	
		24801	Other vegetables	
		24803	Mixtures of two or more vegetables	
		24901	Vegetables and sauce	
		24902	Stuffed vegetables and vegetable dishes	
		24903	Fried vegetable dishes	
	Frozen peas	24501	Peas and edible-podded peas	
		24502	Beans	
		24503	Sprouts	
	Baked Beans, canned	20301	Cheese substitute	
		20601	Meat substitutes	
		20701	Dishes where meat substitutes are the major component	
		25201	Legume and pulse products	
		25202	Dishes where mature legumes are the major component	
**Grain (Cereal) Foods – Wholegrain and Refined**	Weetbix	12511	Breakfast cereal, mixed grain	
		12512	Breakfast cereal, mixed grain, fortified, sugars ≤20 g/100g	
		12514	Breakfast cereal, mixed grain, with fruit and/or nuts	
		12515	Breakfast cereal, mixed grain, with fruit and/or nuts, fortified	
		12516	Breakfast cereal, other	
	Wholemeal bread, pre-packaged	12204	Breads, and bread rolls, mixed grain, mandatorily fortified	
		12205	Breads, and bread rolls, mixed grain, additional voluntary fortification	
		12206	Breads, and bread rolls, mixed grain, not stated as to fortification	
		12207	Breads, and bread rolls, wholemeal and brown, mandatorily fortified	
		12208	Breads, and bread rolls, wholemeal and brown, additional voluntary fortification	
		12209	Breads, and bread rolls, wholemeal, not stated as to fortification	
		12210	Breads, and bread rolls, rye, mandatorily fortified	
		12211	Breads, and bread rolls, rye, additional voluntary fortification	
		12212	Breads, and bread rolls, rye, not stated as to fortification	
		12214	Breads, and bread rolls, not stated as to major flour or fortification	
	Rolled oats	12601	Porridge style, oat based	
		12602	Porridge style, other cereals	
	White bread, pre-packaged	12201	Breads, and bread rolls, white, mandatorily fortified	
		12202	Breads, and bread rolls, white, additional voluntary fortification	
		12203	Breads, and bread rolls, white, not stated as to fortification	

Food Group	Food and drink items of the Low SEG Healthy Diets ASAP habitual diet pricing tool	AHS NNPAS Code	Item	Proportion of weight included (100% if blank)
		12213	Breads, and bread rolls, gluten free	
		12301	English-style muffins	
		12302	Flat breads (e.g. Pita bread), wheat based	
		12303	Flat breads (e.g. Pita bread), other cereal flours	
		13307	Scones and rock cakes, plain or with added fruit or vegetables only	
		13606	Crumpets	
	Cornflakes	12501	Breakfast cereal, corn based	
		12502	Breakfast cereal, corn based, fortified	
		12503	Breakfast cereal, rice based	
		12504	Breakfast cereal, rice based, fortified	
		12505	Breakfast cereal, wheat based	
		12506	Breakfast cereal, wheat based, fortified, sugars ≤20 g/100g	
		12508	Breakfast cereal, wheat based, with fruit and/or nuts, unfortified	
		12509	Breakfast cereal, wheat based, with fruit and/or nuts, fortified, sugars ≤25 g/100g	
	White pasta	12101	Grains (other than rice) and grain fractions	
		12103	Cereal flours and starches	
		12104	Cereal flours and starches, fortified	
		12401	Pasta and noodles, wheat based, other than instant noodles	
		12403	Pasta and noodles, not wheat based	
		12404	Filled pasta	50%
		18708	Pork dishes with gravy, sauce or vegetables	35%
		18709	Pork dishes, added pasta, noodles or rice	35%
		18901	Poultry dishes, with gravy, sauce or vegetables	35%
		18902	Poultry dishes, with gravy, sauce or vegetables, added pasta, noodles or rice	35%
		18801	Sausage dishes with gravy, sauce or vegetables	35%
		18802	Sausage dishes with gravy, sauce or vegetables, added pasta, noodles or rice	35%
	White rice	12102	Rice and rice grain fractions	
		12404	Filled pasta	50%
	Dry wheat crackers, water crackers	13201	Savoury biscuits, wheat based, plain, energy ≤1800 kJ per 100 g	
		13203	Savoury biscuits, rye based	
		13204	Savoury biscuits, rice based (includes rice cakes)	
		13205	Savoury biscuits, corn based	
**Lean Meats and Poultry, Fish, Eggs, Nuts and Seeds**	Beef mince, lean	18103	Pork	
		18104	Veal	
		18201	Kangaroo	
		18202	Other mammalian game	
		18401	Liver	
		18402	Kidney	
	Lamb loin chops	18102	Lamb and mutton	
		18708	Pork dishes with gravy, sauce or vegetables	50%
		18709	Pork dishes, added pasta, noodles or rice	50%
	Beef rump steak	18101	Beef	
	Tuna, canned in oil	15101	Fin fish, fresh, frozen	
		15102	Smoked fish	
		15201	Crustacea, fresh, frozen	
		15202	Molluscs, fresh, frozen	
		15301	Fish roe	
		15302	Eel	
		15303	Mixtures of finfish and/or shellfish	
		15401	Packed fin fish	
		15402	Packed crustacea and molluscs	
		15504	Fish and seafood products	
		15601	Mixed dishes with fish as the major component	
		15602	Mixed dishes with fish as the major component, with rice, pasta or noodles	
		15603	Mixed seafood dishes with crustacea, molluscs or other seafood products as the major component	
		15604	Mixed seafood dishes with crustacea, molluscs or other seafood products as the major component, with rice, pasta or noodles	
	Chicken, cooked whole	18301	Chicken	
		18302	Other poultry	
		18303	Feathered game	
		18901	Poultry dishes, with gravy, sauce or vegetables	50%
		18902	Poultry dishes, with gravy, sauce or vegetables, added pasta, noodles or rice	50%
	Eggs	17101	Eggs, chicken	
		17102	Eggs, chicken, modified (e.g. Omega-3, folate)	
		17103	Eggs, other	
		17201	Egg dishes, savoury	
		17202	Egg dishes, sweet	
		24905	Salads, vegetable based, added meat, fish or eggs	50%
	Canned meat and vegetable casserole	18701	Beef dishes with gravy, sauce or vegetables	
		18702	Beef dishes, added pasta, noodles or rice	
		18705	Lamb or mutton dishes with gravy, sauce or vegetables	
		18706	Lamb or mutton dishes, added pasta, noodles or rice	
**Milk, Yoghurt, Cheese and Alternatives**	Cheddar cheese, full fat	19401	Cheese, hard cheese ripened styles	
		19403	Cheese, unripened styles, including cream and cottage cheese, regular fat	
		19405	Cheese, camembert, brie and other surface ripened cheeses	
		19406	Cheese, processed	
		19408	Cheese, not further defined	
	Milk, full fat	19101	Milk, cow, fluid, regular whole, full fat	
		19102	Milk, cow, fluid, regular whole, full fat, fortified	
		19106	Milk, evaporated or condensed, undiluted	
		19107	Milk, powder, cow, dry	
		19108	Milk, non-bovine species	
		19109	Milk, fluid, unspecified	
	Cheddar cheese, reduced fat	19402	Cheese, hard cheese ripened styles, reduced fat	
		19404	Cheese, unripened styles, including cream and cottage cheese, reduced fat	
		19407	Cheese, processed, reduced fat	
	Milk, reduced fat	19103	Milk, cow, fluid, reduced fat, <2 g/100g	
		19104	Milk, cow, fluid, reduced fat, <2 g/100g, fortified	
		19105	Milk, cow, fluid, skim, non-fat	
	Yoghurt, full fat, plain	19201	Yoghurt, natural, regular fat and high fat (>4 g/100g fat)	
		19202	Yoghurt, natural, reduced fat	
		19203	Yoghurt, natural, skim and non-fat	
	Yoghurt, flavoured reduced fat	19204	Yoghurt, flavoured or added fruit and/or cereal, high fat (>4 g/100g fat)	
		19205	Yoghurt, flavoured or added fruit, full fat	
		19206	Yoghurt, flavoured or added fruit with added cereal, full fat	
		19207	Yoghurt, flavoured or added fruit, reduced fat	
		19208	Yoghurt, flavoured or added fruit, low fat or skim, sugar sweetened	

Food Group	Food and drink items of the Low SEG Healthy Diets ASAP habitual diet pricing tool	AHS NNPAS Code	Item	Proportion of weight included (100% if blank)
		19209	Yoghurt, flavoured or added fruit, low fat or skim, intense sweetened	
		19210	Yoghurt, drinks, buttermilk	
		19211	Yoghurt, added nutrients or other substances	
		19212	Yoghurt, unspecified fat	
		20502	Soy-based yoghurts, reduced fat	
**Unsaturated Oils and Spreads (or foods from which these are derived)**	Canola margarine	14301	Polyunsaturated margarine spreads, fat content ≥ 65g/100g	
		14302	Polyunsaturated margarine spreads, fat content <65 g/100g	
		14303	Monounsaturated margarine spreads, fat content ≥65 g/100g	
		14304	Monounsaturated margarine spreads,fat content <65 g/100g	
		14305	Cooking margarine	
		14306	Margarine spreads with added phytosterols	
		14307	Unspecified margarine spread	
		14403	Unspecified vegetable/nut oils	
		14502	Vegetable-based solid fats	
	Sunflower oil	14401	Polyunsaturated oils	50%
		14402	Monounsaturated oils	50%
	Olive oil	14401	Polyunsaturated oils	50%
		14402	Monounsaturated oils	50%
**Pre-prepared mixed foods**	Sandwich, pre-made, white bread, chicken, salad, cheese	13503	Sandwiches and filled rolls, saturated fat ≤5 g/100 g	
**Discretionary Choices**	Beer	29101	Beers, > 3.5% alcohol	
		29102	Beers, 1.15- 3.5% alcohol, reduced alcohol / light	
		29401	Cider	
		29402	Perry	
	White wine, sparkling	29202	Wines, white (including sparkling varieties)	
		29203	Fortified wines	
		29204	Reduced alcohol wines	
	Whisky	29301	Spirits	
		29501	Liqueurs	
		29502	Cocktails and other mixed drinks	25%
		29503	Pre-mixed drinks, cola- or energy-drink based	25%
		29504	Pre-mixed drinks, other	25%
	Red wine	29201	Wines, red (including sparkling varieties and rose styles)	
	Butter	14101	Butter	
		14102	Butter products	
		14201	Dairy blend, regular, fat content ≥ 65g/100g	
		14202	Dairy blend, reduced fat, fat content <65 g/100g	
		14203	Dairy fats, unspecified type	
		14501	Animal-based solid fats	
		14503	Other fats or oils	
		14601	Unspecified dairy-based fat or margarine used as a spread	
		14602	Unspecified fats or oil used in cooking	
		19301	Cream, regular and increased fat	
		19302	Cream, reduced fat	
		19303	Cream, sour	
		19304	Cream, sour, reduced fat	
		19305	Cream substitute, artificial cream	
		19306	Cream, unspecified type or fat level	
	Muffin, commercial	12305	Sweet breads, buns and scrolls, uniced, unfilled	
		12306	Sweet breads, buns and scrolls, iced and/or filled	
		13301	Cakes and cake mixes, chocolate	
		13302	Cakes and cake mixes, sponge	
		13303	Cakes and cake mixes, other types	
		13304	Muffins, cake type, and muffin mixes	
		13305	Cake-type desserts	
		13306	Slices, biscuit and cake-type	
		13309	Other desserts containing cereal	
		13601	Pancakes, crepes and dishes	
		13602	Drop scones, pikelets	
		13603	Waffles	
		13604	Batters and batter puddings	
		13605	Doughnuts	
	Cream-filled sweet biscuit, pre-packaged	13101	Sweet biscuits, plain or flavoured including short bread varieties	
		13102	Sweet biscuits, plain with fruit or nuts	
		13103	Sweet biscuits, with jam, marshmallow or other sugar-based filling	
		13104	Sweet biscuits, cream-filled	
		13105	Sweet biscuits, chocolate-coated, chocolate chip	
		13106	Sweet biscuits, chocolate-coated, chocolate or cream filled	
		13107	Sweet biscuits, other toppings	
		13402	Sweet pastry products, fruit and/or nut fillings	
		13403	Sweet pastry products, egg or dairy based fillings	
	Muesli bar, pre-packaged	12507	Breakfast cereal, wheat based, fortified, sugars >20 g/100g	
		12510	Breakfast cereal, wheat based, with fruit and/or nuts, fortified, sugars >25 g/100g	
		12513	Breakfast cereal, mixed grain, fortified, sugars >20 g/100g	
		28201	Fruit bar and fruit-based confectionery	
		28202	Nut and seed based confectionery	
		28301	Muesli and cereal style bars, no fruit	
		28302	Muesli and cereal style bars, with fruit and/or nuts	
		28303	Muesli and cereal style bars, added coatings or confectionery	
		28304	Muesli bar, with fruit or fruit paste filling	
		28305	Snack bar, other	
		30101	Biscuit and bar meal replacement	
	Nuts, mixed, salted	16803	Dried fruit and nut mixes	
		22101	Seeds	
		22102	Seed products	
		22201	Peanuts	
		22202	Peanut products	
		22203	Coconut and coconut products	
		22204	Other nuts and nut products and dishes	
		22205	Mixed nuts or nuts and seeds	
		22301	Wild harvested seeds	
		22302	Wild harvested nuts	
	Pizza	12304	Savoury filled or topped breads and bread rolls	
		12307	Fried bread products and garlic breads	
		13308	Scones and rock cakes, with added cheese, chocolate or similar	
		13501	Pizza, saturated fat ≤5 g/100 g	
		13502	Pizza, saturated fat >5 g/100 g	
	Savoury flavoured biscuits	13202	Savoury biscuits, wheat based, plain, energy >1800 kJ per 100 g	
		13401	Pastry, plain/unfilled, all types	

Food Group	Food and drink items of the Low SEG Healthy Diets ASAP habitual diet pricing tool	AHS NNPAS Code	Item	Proportion of weight included (100% if blank)
	Confectionary	11801	Fortified beverage flavourings prepared with water or milk	
		11802	Fortified dry beverage flavourings	
		11803	Unfortified beverage flavourings prepared with water or milk	
		11804	Unfortified dry beverage flavourings	
		28401	Lollies and other confectionery, sugar sweetened	
		28402	Lollies and other confectionery, intense sweetened	
		28403	Chewing gum, sugar sweetened	
		28404	Chewing gum, artificially sweetened	
		28405	Other confectionery	
	Chocolate	28101	Chocolate (plain, unfilled varieties)	
		28102	Chocolate-based confectionery with nut fillings or additions	
		28103	Chocolate-based confectionery with other fillings or additions	
	Sugar-sweetened beverages (Coca Cola)	11401	Cordials, made from concentrate	
		11402	Cordials, made from concentrate, intense sweetened	
		11403	Cordial concentrate	
		11404	Cordial concentrate, intense sweetened	
		11501	Soft drinks, non-cola	
		11503	Soft drinks, cola	
		11505	Flavoured mineral waters	
		11601	Electrolyte drinks (sports drinks)	
		11602	Electrolyte drink bases (sport drink bases)	
		11603	Energy drinks	
		29205	De-alcoholised and non-alcoholic wine (including sparkling varieties)	
		29502	Cocktails and other mixed drinks	75%
		29503	Pre-mixed drinks, cola- or energy-drink based	75%
		29504	Pre-mixed drinks, other	75%
	Artificially sweetened beverages	11502	Soft drinks, non-cola, intense sweetened	
		11504	Soft drinks, cola, intense sweetened	
		11506	Flavoured mineral waters, intense sweetened	
		11604	Energy drinks, intense sweetened	
	Flavoured milk	11805	Breakfast cereal beverages	
		11806	Other beverages	
		19801	Milk, coffee/chocolate flavoured and milk-based drinks, full fat	
		19802	Milk, other flavoured and milk-based drinks, full fat	
		19803	Milk, coffee/chocolate flavoured and milk-based drinks, reduced fat	
		19804	Milk, other flavoured and milk-based drinks, reduced fat	
		19805	Milk, other flavoured and milk-based drinks, not stated as to fat	
		19806	Milk-based fruit drinks	
		20101	Soy-based beverage, plain	
		20102	Soy-based beverage, plain, fortified	
		20103	Soy-based beverage, plain, reduced fat	
		20104	Soy-based beverage, plain, reduced fat, fortified	
		20105	Soy-based beverage, plain, skim, fortified	
		20106	Cereal- or nut-based milk substitute	
		20107	Dairy milk substitute, unspecified	
		20201	Soy-based beverage, regular fat, flavoured	
		20202	Soy-based beverage, reduced fat, flavoured	
		30102	Meal replacement and similar prepared beverages	
		30103	Meal replacement and similar dry powders	
		30104	Sport and protein prepared beverages	
		30105	Sport and protein, dry powders	
		30106	Supplementary and medical foods prepared beverages	
		30107	Supplementary and medical foods dry powders	
	Meat pie, commercial	13404	Savoury pastry products, quiches and flans	
		13405	Savoury pastry products, pies, rolls and envelopes	
		13406	Savoury pastry products, pies, rolls and envelopes, fried	
		13507	Taco and tortilla-based dishes, saturated fat ≤5 g/100 g	
		13508	Taco and tortilla-based dishes, saturated fat >5 g/100 g	
	Frozen lasagne, pre-packaged	13509	Savoury pasta/noodle and sauce dishes, saturated fat ≤5 g/100 g	
		13510	Savoury pasta/noodle and sauce dishes, saturated fat >5 g/100 g	
		13511	Savoury rice-based dishes, saturated fat ≤5 g/100 g	
		13512	Savoury rice-based dishes, saturated fat >5 g/100 g	
		13513	Savoury dumplings	
		13514	Sushi, all types	
		13515	Other savoury grain dishes	
	Hamburger, commercial	13504	Sandwiches and filled rolls, saturated fat >5 g/100 g	
		13505	Burgers, saturated fat ≤5 g/100 g	
		13506	Burgers, saturated fat >5 g/100 g	
		18703	Beef, crumbed, battered, meatloaf or patty type with cereal and/or vegetables	
		18704	Veal, crumbed, battered, meatloaf or patty type with cereal and/or vegetables	
		18707	Lamb or mutton, crumbed, battered, meatloaf or patty type with cereal and/or vegetables	
		18710	Pork, crumbed, battered, meatloaf or patty type with cereal and/or vegetables	
		18903	Poultry crumbed, battered, meatloaf or patty type with cereal and/or vegetables	
	Beef sausages	18403	Other organ meats and offal	
		18404	Liver paste, pate and dishes	
		18405	Other organ meat, blood and offal dishes	
		18501	Sausage, saturated fat content >5 g/100g	
		18502	Frankfurts and saveloys, saturated fat content >5 g/100g	
		18503	Sausages, frankfurts and saveloys, saturated fat content ≤5 g/100g	
		18601	Bacon	
		18603	Fermented, comminuted meats (e.g. Salami)	
		18604	Processed delicatessen meat, mammalian	
		18605	Processed delicatessen meat, poultry	
		18606	Processed meat, commercially sterile (includes canned meats)	
		18607	Dried meats	
		18711	Mammalian game meat, mixed dishes	
		18712	Unspecified meat, mixed dishes	
		18801	Sausage dishes with gravy, sauce or vegetables	50%
		18802	Sausage dishes with gravy, sauce or vegetables, added pasta, noodles or rice	50%
		18803	Other processed meat, mixed dish	
	Ham	18602	Ham	
	Potato crisps, pre-packaged	26101	Potato crisps	
		26102	Other vegetable crisps	
		26201	Corn chips	
		26202	Popcorn	
		26301	Extruded snacks	
		26401	Other snacks	

Food Group	Food and drink items of the Low SEG Healthy Diets ASAP habitual diet pricing tool	AHS NNPAS Code	Item	Proportion of weight included (100% if blank)
	Potato chips, commercial	24102	Potato products	
	Ice cream	19501	Ice cream, tub varieties, fat content >10 g/100 g	
		19502	Ice cream, tub varieties, fat content 4 - 10 g/100 g	
		19503	Ice cream, tub varieties, fat content <4 g/100 g	
		19504	Ice cream, individual bar, stick and cone varieties, fat content >10 g/100 g	
		19505	Ice cream, individual bar, stick and cone varieties, fat content 4 - 10 g/100 g	
		19506	Ice cream, individual bar, stick and cone varieties, fat content <4 g/100 g	
		19507	Frozen yoghurts, all types	
		19508	Frozen dairy desserts, other	
		19601	Custard, fat content ≥ 4 g/100 g	
		19602	Custard, fat content <4 g/100 g	
		19701	Dairy desserts, smooth or gelatin-based dairy desserts	
		19702	Other milk, cheese or cream-based desserts	
		20401	Soy-based ice confection	
		27303	Water ice confection, gelato, sorbet	
	White sugar	27101	Sugar	
		27102	Honey and sugar syrups	
		27103	Toppings, all flavours	
		27201	Jams and conserves, sugar sweetened	
		27202	Jams and conserves, reduced sugar	
		27203	Sweet spreads, fruit flavoured	
		27204	Sweet spreads or sauces, chocolate/coffee flavoured	
		27205	Sauces, sweet, fruit-based	
		27301	Sugar-based desserts	
		27302	Sugar-based desserts, intense sweetened	
	Salad dressing	23301	Mayonnaise and cream-style dressings, full fat	
		23302	Mayonnaise and cream-style dressings, reduced or non-fat	
		23303	Italian and French-style dressings, full fat	
		23304	Italian and French-style dressings, reduced or non-fat	
		23305	Vinegar	
		23401	Bread-based stuffings	
		23501	Dairy based dips	
		23502	Vegetable based dips	
		23503	Legume based dips	
		23504	Other dips	
	Tomato sauce	23101	Gravies (prepared)	
		23102	Dry gravy mixes	
		23103	Savoury sauces, not tomato based, commercial	
		23104	Savoury sauces, tomato based, commercial	
		23105	Savoury sauces, not tomato based, homemade	
		23106	Savoury sauces, tomato based, homemade	
		23107	Savoury sauces, commercial, simmer style	
		23108	Savoury sauces, dairy based, homemade	
		23109	Savoury pastes	
		23110	Dry savoury sauces and casserole bases and dry mixes	
		23201	Fruit-based pickles, chutneys and relishes	
		23202	Vegetable-based pickles, chutneys and relishes	
	Chicken soup, canned	21101	Soup containing meat, poultry or seafood	
		21102	Soup, vegetable only	
		21201	Dry soup mix containing meat, poultry or seafood	
		21202	Dry soup mix, vegetable only	
		21301	Soup containing meat, poultry or seafood	
		21302	Soup, vegetable only	
		21401	Soup containing meat, poultry or seafood	

Food Group	Food and drink items of the Low SEG Healthy Diets ASAP habitual diet pricing tool	AHS NNPAS Code	Item	Proportion of weight included (100% if blank)
		21402	Soup, vegetable only	
		21501	Soup containing meat, poultry or seafood	
		21502	Soup, vegetable only	
		21601	Soup containing meat, poultry or seafood	
		21602	Soup, vegetable only	
		31304	Stock, prepared	
	Orange fruit juice	11301	Fruit juices, commercially prepared	
		11302	Fruit juices, freshly-squeezed	
		11303	Fruit juices, fortified	
		11304	Vegetable juices	
		11305	Vegetable juices, freshly squeezed	
		11306	Fruit and vegetable juice blends	
		11307	Fruit drinks (ready to drink or made from concentrate)	
		11308	Vegetable drinks	
	Fish fillet crumbed, pre-packaged	15501	Fin fish, battered or crumbed	
		15502	Crustacea, battered or crumbed	
		15503	Molluscs, battered or crumbed	
	Instant noodles, wheat based	12402	Instant noodles and noodle products, wheat based	
**Items not included**	Items not included	11101	Tea, regular, caffeinated, prepared with water	
		11102	Tea, regular, caffeinated, prepared with milk or milk substitute	
		11103	Tea, regular, decaffeinated, prepared with water or milk	
		11104	Tea mixed with other foods	
		11105	Herbal tea	
		11106	Tea powders and bases	
		11201	Coffee beverage, prepared with water	
		11202	Coffee beverage, prepared with milk or milk substitute	
		11203	Coffee beverage, decaffeinated, prepared with water	
		11204	Coffee beverage, decaffeinated, prepared with milk or milk substitute	
		11205	Dry coffee powder, caffeinated or decaffeinated	
		11206	Coffee substitutes, beverage	
		11207	Coffee substitutes, powders and bases	
		11208	Coffee-based mixes, beverage	
		11209	Dry or concentrate coffee-based mixes	
		11308	Vegetable drinks	
		11309	Fruit drink, prepared from dry powder	
		11701	Domestic water (including tap, tank/rain water)	
		20501	Soy-based yoghurts, regular fat	
		20502	Soy-based yoghurts, reduced fat	
		24001	Wild harvested vegetables	
		24002	Wild harvested vegetable dishes	
		27304	Frostings and icing	
		28104	Carob or yoghurt and carob or yoghurt-based confectionery	
		29505	Other alcoholic beverages	
		31101	Yeast	
		31102	Yeast extracts	
		31103	Vegetable and meat extracts	
		31201	Intense sweeteners	
		31301	Salt	
		31302	Herbs(dried) and spices	
		31303	Stock cubes and seasonings	
		31401	Essences	
		31501	Chemical raising agents and cooking ingredients	
		31502	Gelatine	
		31503	Other additives	
		32101	Infant formula, prepared	

Food Group	Food and drink items of the Low SEG Healthy Diets ASAP habitual diet pricing tool	AHS NNPAS Code	Item	Proportion of weight included (100% if blank)
		32102	Human breast milk	
		32103	Toddler formula, prepared	
		32201	Infant cereals	
		32202	Infant rusks	
		32203	Infant pasta and rice dishes	
		32302	Infant savoury dishes, commercial	
		32303	Infant custards or yoghurts	
		32401	Infant fruit juices	
		34101	Reptiles	
		34201	Insects	
		34301	Amphibia	
