# Supplementary material for: Dietary Intake, Cost, and Affordability by Socioeconomic Group in Australia
Source: Int J Environ Res Public Health. 2021 Dec 17;18(24):13315. doi: 10.3390/ijerph182413315 (PMC8703846; doi:10.3390/ijerph182413315)
Supplement: Supplementary file 1 [file ijerph-18-13315-s001.zip › Additional table 2.pdf]

Table S2: Composition of the habitual diet of each SEG, and the recommended diet, for the reference household (two adults, two children)

| | **Habitual Diet** | | | | | |
|---|---|---|---|---|---|---|
| **Food item** | **SEG Quintile 1 (lowest income)** | **SEG Quintile 2** | **SEG Quintile 3** | **SEG Quintile 4** | **SEG Quintile 5 (highest income)** | **Recommended Diet** |
| **Energy (kJ/day)** | **32,517 kJ** | **34,182 kJ** | **33,279 kJ** | **34,390 kJ** | **34,862 kJ** | **32,996 kJ** |
| **Water** | | | | | | |
| Water, bottled (mL/fortnight) | 3485 | 5126 | 5379 | 4527 | 7714 | 5296 |
| **Fruit** | | | | | | |
| Apples (g/fortnight) | 3638 | 2974 | 2824 | 3203 | 3194 | 5460 |
| Bananas (g/fortnight) | 795 | 1069 | 1056 | 892 | 1158 | 5460 |
| Oranges (g/fortnight) | 971 | 1291 | 1752 | 1354 | 1261 | 5460 |
| Fruit salad, canned in juice (g/fortnight) | 1544 | 2335 | 2820 | 2682 | 2443 | 0 |
| *Total fruit (g/fortnight)* | *9614* | *10214* | *11495* | *11086* | *11077* | *16380* |
| **Vegetables and legumes** | | | | | | |
| Potato, loose (g/fortnight) | 1844 | 1863 | 1597 | 1379 | 1650 | 2320 |
| Broccoli, loose (g/fortnight) | 389 | 408 | 259 | 431 | 632 | 1470 |
| White cabbage, loose (g/fortnight) | 175 | 254 | 238 | 216 | 296 | 1470 |
| Iceberg lettuce, whole (g/fortnight) | 704 | 614 | 861 | 1041 | 1059 | 1470 |
| Carrot, loose (g/fortnight) | 618 | 758 | 839 | 593 | 650 | 2205 |
| Pumpkin (g/fortnight) | 330 | 301 | 253 | 299 | 265 | 2205 |
| Onion, loose (g/fortnight) | 106 | 104 | 108 | 88 | 151 | 1638 |
| Tomatoes, loose (g/fortnight) | 654 | 465 | 552 | 477 | 474 | 1638 |
| Sweetcorn, canned (g/fortnight) | 216 | 265 | 231 | 292 | 479 | 1160 |
| Four bean mix, canned (g/fortnight) | 61 | 54 | 59 | 106 | 68 | 1005 |
| Diced tomatoes, canned (g/fortnight) | 175 | 193 | 190 | 306 | 302 | 1638 |
| Baked Beans, canned (g/fortnight) | 237 | 1119 | 321 | 407 | 420 | 1005 |
| Frozen mixed vegetables (g/fortnight) | 746 | 1187 | 1703 | 1080 | 1308 | 1638 |
| Frozen peas (g/fortnight) | 334 | 351 | 201 | 328 | 359 | 1638 |
| *Total vegetables and legumes (g/fortnight)* | *7136* | *8620* | *8332* | *7765* | *8840* | *22620* |
| **Grain (cereal) foods, mostly wholegrain** | | | | | | |
| Wholemeal bread, pre-packaged (g/fortnight) | 870 | 1020 | 1101 | 1265 | 1205 | 4272 |
| White bread, pre-packaged (g/fortnight) | 3001 | 2785 | 2713 | 2532 | 2505 | 893 |
| Rolled oats (g/fortnight) | 578 | 959 | 724 | 1155 | 689 | 6648 |
| Breakfast cereal, corn flakes (g/fortnight) | 509 | 612 | 668 | 774 | 809 | 670 |
| Breakfast cereal, wheat biscuits (g/fortnight) | 243 | 222 | 234 | 606 | 560 | 2216 |
| White pasta (g/fortnight) | 988 | 864 | 1380 | 1047 | 1590 | 2042 |
| White rice (g/fortnight) | 1904 | 1820 | 2284 | 1450 | 1338 | 2042 |
| Dry wheat crackers, water crackers (g/fortnight) | 89 | 139 | 219 | 154 | 177 | 781 |
| *Total grain (cereal) foods (g/fortnight)* | *8336* | *8616* | *9506* | *9111* | *9042* | *19684* |
| **Lean meats, poultry, fish, eggs, and plant-based alternatives** | | | | | | |
| Tuna, canned in oil (g/fortnight) | 760 | 725 | 849 | 590 | 739 | 1841 |
| Beef mince, lean (g/fortnight) | 163 | 306 | 259 | 390 | 285 | 1168 |
| Lamb loin chops (g/fortnight) | 333 | 335 | 423 | 409 | 709 | 1169 |
| Beef rump steak (g/fortnight) | 1042 | 866 | 1095 | 733 | 1201 | 1172 |
| Eggs (g/fortnight) | 884 | 828 | 743 | 982 | 1051 | 2208 |
| Chicken, cooked whole (g/fortnight) | 1093 | 2073 | 1721 | 1966 | 2208 | 1471 |
| Peanuts, roasted, unsalted (g/fortnight) | 0 | 0 | 0 | 0 | 0 | 780 |
| *Total lean meats, poultry, fish, eggs, and plant-based* | *4822* | *5816* | *6007* | *5793* | *6919* | *9929* |

| Food item | Habitual Diet SEG Quintile 1 (lowest income) | SEG Quintile 2 | SEG Quintile 3 | SEG Quintile 4 | SEG Quintile 5 (highest income) | Recommended Diet |
|---|---|---|---|---|---|---|
| *alternatives (g/fortnight)* | | | | | | |
| **Milk, yoghurt, cheese, and plant-based alternatives** | | | | | | |
| Cheddar cheese, full fat (g/fortnight) | 682 | 529 | 601 | 606 | 772 | 704 |
| Cheddar cheese, reduced fat (g/fortnight) | 49 | 112 | 81 | 90 | 82 | 516 |
| Milk, full fat (mL/fortnight) | 7301 | 6850 | 6229 | 5367 | 4527 | 6438 |
| Milk, reduced fat (mL/fortnight) | 1839 | 2672 | 2868 | 3785 | 4384 | 12000 |
| Flavoured milk (mL/fortnight) | 2187 | 3108 | 2807 | 2066 | 2539 | 0 |
| Yoghurt, full fat, plain (g/fortnight) | 101 | 355 | 202 | 205 | 242 | 2576 |
| Yoghurt, flavoured reduced fat (g/fortnight) | 722 | 1151 | 1107 | 973 | 1361 | 5100 |
| *Total milk, yoghurt, cheese, and plant-based alternatives (g/fortnight)* | *12880* | *14776* | *13895* | *13092* | *13907* | *27334* |
| **Unsaturated oils and spreads** | | | | | | |
| Sunflower oil (mL/fortnight) | 15 | 11 | 10 | 9 | 12 | 291 |
| Olive oil (mL/fortnight) | 15 | 11 | 10 | 9 | 12 | 291 |
| Canola margarine (g/fortnight) | 197 | 166 | 131 | 166 | 120 | 412 |
| *Total unsaturated oils and spreads (g/fortnight)* | *226* | *189* | *152* | *184* | *144* | *994* |
| **Discretionary choices - other** | | | | | | |
| Chicken soup, canned (g/fortnight) | 2219 | 1984 | 1656 | 1387 | 1796 | 0 |
| Muffin, commercial (g/fortnight) | 922 | 1767 | 1366 | 1709 | 1562 | 0 |
| Instant noodles, wheat based (g/fortnight) | 227 | 464 | 361 | 1347 | 304 | 0 |
| White sugar (g/fortnight) | 714 | 690 | 546 | 616 | 646 | 0 |
| Cream-filled sweet biscuit, pre-packaged (g/fortnight) | 628 | 709 | 586 | 511 | 644 | 0 |
| Muesli bar, pre-packaged (g/fortnight) | 339 | 383 | 371 | 374 | 390 | 0 |
| Savoury flavoured biscuits (g/fortnight) | 207 | 306 | 267 | 286 | 306 | 0 |
| Nuts, mixed, salted (g/fortnight) | 262 | 132 | 332 | 245 | 304 | 0 |
| Confectionary (g/fortnight) | 396 | 501 | 368 | 359 | 274 | 0 |
| Chocolate (g/fortnight) | 359 | 342 | 426 | 343 | 419 | 0 |
| Potato crisps, pre-packaged (g/fortnight) | 650 | 503 | 554 | 656 | 333 | 0 |
| Salad dressing (mL/fortnight) | 211 | 222 | 371 | 408 | 297 | 0 |
| Tomato sauce (g/fortnight) | 511 | 626 | 784 | 503 | 558 | 0 |
| Beef sausages (g/fortnight) | 1036 | 1001 | 1462 | 1212 | 1114 | 0 |
| Butter (g/fortnight) | 195 | 173 | 226 | 154 | 202 | 0 |
| Ham (g/fortnight) | 143 | 175 | 220 | 185 | 217 | 0 |
| Frozen lasagne, pre-packaged (g/fortnight) | 3684 | 2590 | 3346 | 4369 | 4352 | 0 |
| Fish fillet crumbed, pre-packaged (g/fortnight) | 433 | 418 | 264 | 503 | 194 | 0 |
| Ice cream (g/fortnight) | 1307 | 1388 | 1393 | 1636 | 1294 | 0 |
| *Total discretionary choices – other (g/fortnight)* | *17106* | *16919* | *17941* | *19759* | *18228* | *0* |
| **Alcohol** | | | | | | |
| Beer, full strength (mL/fortnight) | 5060 | 4857 | 4903 | 5053 | 5796 | 0 |
| White wine, sparkling (mL/fortnight) | 546 | 418 | 799 | 845 | 1443 | 0 |
| Whisky (mL/fortnight) | 453 | 378 | 227 | 281 | 340 | 0 |
| Red wine (mL/fortnight) | 519 | 668 | 561 | 1193 | 1971 | 0 |
| *Total alcohol* (mL/fortnight) | *6579* | *6321* | *6490* | *7372* | *9550* | *0* |
| **Takeaway foods** | | | | | | |
| Pizza, commercial (g/fortnight) | 1800 | 1084 | 849 | 1264 | 1770 | 0 |

| | Habitual Diet | | | | | |
|---|---|---|---|---|---|---|
| **Food item** | **SEG Quintile 1 (lowest income)** | **SEG Quintile 2** | **SEG Quintile 3** | **SEG Quintile 4** | **SEG Quintile 5 (highest income)** | **Recommended Diet** |
| Meat pie, commercial (g/fortnight) | 1554 | 1533 | 1840 | 1512 | 1554 | 0 |
| Hamburger, commercial (g/fortnight) | 2710 | 3421 | 2324 | 2448 | 2145 | 0 |
| Potato chips, commercial (g/fortnight) | 833 | 1649 | 737 | 977 | 658 | 0 |
| *Total takeaway foods (g/fortnight)* | *6897* | *7687* | *5751* | *6201* | *6126* | *0* |
| **Sugar sweetened beverages** | | | | | | |
| Sugar sweetened beverages (mL/fortnight) | 16288 | 11781 | 12034 | 12865 | 10896 | 0 |
| **Artificially sweetened beverages** | | | | | | |
| Artificially sweetened beverages (mL/fortnight) | 1406 | 1883 | 2503 | 2772 | 3146 | 0 |
| **Items divided between more than one food group** | | | | | | |
| Sandwich, pre-made, white bread, chicken, and salad* (g/fortnight) | 462 | 586 | 547 | 387 | 510 | 360 |
| Canned meat and vegetable casserole** (g/fortnight) | 786 | 975 | 1471 | 1188 | 1112 | 0 |
| Orange fruit juice*** (mL/fortnight) | 5331 | 5093 | 6084 | 5910 | 6043 | 0 |

*Divided equally between Grains etc, Lean meats etc, and Vegetables
**Divided equally between Lean meats etc and Vegetables
***Divided equally between Fruit and Discretionary choices - other
