# Supplementary material for: Dietary Intake, Cost, and Affordability by Socioeconomic Group in Australia
Source: Int J Environ Res Public Health. 2021 Dec 17;18(24):13315. doi: 10.3390/ijerph182413315 (PMC8703846; doi:10.3390/ijerph182413315)
Supplement: Supplementary file 1 [file ijerph-18-13315-s001.zip › Additional table 3.pdf]

Table S3: Habitual diet costs of each SEG and recommended diet costs, for the representative household (two adults, two children)

	Habitual Diet (AUD$/fortnight)					Recommended Diet (AU$/fortnight)
	SEG Quintile 1 (lowest income)	SEG Quintile 2	SEG Quintile 3	SEG Quintile 4	SEG Quintile 5 (highest income)	All Quintiles
**Water**	$12.34	$18.15	$19.05	$16.03	$27.32	$18.76
**Fruit**	$45.66	$50.43	$56.76	$55.13	$53.83	$72.83
**Vegetables and legumes**	$38.83	$45.82	$47.51	$43.42	$49.18	$101.36
**Grain (cereal) foods, mostly wholegrain**	$36.70	$41.06	$42.42	$43.66	$42.93	$108.10
**Lean meats, poultry, fish, eggs and plant-based Alternatives**	$93.34	$96.96	$109.52	$94.14	$127.28	$203.88
**Milk, yoghurt, cheese and plant-based alternatives**	$49.09	$57.29	$54.76	$50.42	$59.50	$113.77
**Unsaturated oils and spreads**	$1.56	$1.29	$1.05	$1.25	$1.01	$7.83
**Artificially sweetened beverages**	$3.54	$4.74	$6.31	$6.99	$7.93	-
**Discretionary choice – other**	$166.11	$160.90	$171.97	$195.18	$175.83	-
**Alcohol**	$90.36	$85.12	$80.48	$97.44	$130.95	-
**Takeaway foods**	$172.15	$203.35	$141.28	$157.10	$150.16	-
**Sugar sweetened beverages**	$41.05	$29.69	$30.32	$32.42	$27.46	-
**Total Healthy food and drinks**	$281.06	$315.74	$337.38	$311.03	$368.98	$626.52
**Total Discretionary food and drinks**	$469.67	$479.07	$424.05	$482.13	$484.39	-
**Total**	**$750.72**	**$794.81**	**$761.43**	**$793.17**	**$853.37**	**$626.52**
